# Characterization of human small heat shock protein HSPB1 α-crystallin domain localized mutants associated with hereditary motor neuron diseases

**DOI:** 10.1038/s41598-017-18874-x

**Published:** 2018-01-12

**Authors:** Stephen D. Weeks, Lydia K. Muranova, Michelle Heirbaut, Steven Beelen, Sergei V. Strelkov, Nikolai B. Gusev

**Affiliations:** 1 Laboratory for Biocrystallography, Department of Pharmaceutical and Pharmacological Sciences, KU Leuven, 3000 Leuven, Belgium; 2 Department of Biochemistry, School of Biology, Moscow State University, Moscow, 119991 Russian Federation

## Abstract

Congenital mutations in human small heat shock protein HSPB1 (HSP27) have been linked to Charcot-Marie-Tooth disease, a commonly occurring peripheral neuropathy. Understanding the molecular mechanism of such mutations is indispensable towards developing future therapies for this currently incurable disorder. Here we describe the physico-chemical properties of the autosomal dominant HSPB1 mutants R127W, S135F and R136W. Despite having a nominal effect on thermal stability, the three mutations induce dramatic changes to quaternary structure. At high concentrations or under crowding conditions, the mutants form assemblies that are approximately two times larger than those formed by the wild-type protein. At low concentrations, the mutants have a higher propensity to dissociate into small oligomers, while the dissociation of R127W and R135F mutants is enhanced by MAPKAP kinase-2 mediated phosphorylation. Specific differences are observed in the ability to form hetero-oligomers with the homologue HSPB6 (HSP20). For wild-type HSPB1 this only occurs at or above physiological temperature, whereas the R127W and S135F mutants form hetero-oligomers with HSPB6 at 4 °C, and the R136W mutant fails to form hetero-oligomers. Combined, the results suggest that the disease-related mutations of HSPB1 modify its self-assembly and interaction with partner proteins thus affecting normal functioning of HSPB1 in the cell.

## Introduction

Inherited peripheral neuropathies are a genetically diverse group of neurodegenerative disorders that affect the function and survival of motor or sensory neurons in the peripheral nervous system^1,2^. Amongst this clinically heterogeneous group, Charcot-Marie-Tooth (CMT) disease is the most common, with a reported incidence of 1 in 2500 individuals^2^. Patients with this disease demonstrate progressive degeneration of the foot, lower leg, hand and forearm muscles and also suffer from distal sensory loss^2,3^. Presently, mutations in more than 40 highly diverse proteins have been linked to the development of the CMT disease or related distal hereditary neuropathies^2–4^. These proteins include HSPB1 (also known as HSP27), HSPB3 and HSPB8 (HSP22), three members of the molecular chaperone family called the small heat shock proteins (sHSPs)^1,5^.

sHSPs contribute to the protein quality control system and are involved in many vital cellular processes^6–8^. These ATP-independent chaperones prevent the accumulation of improperly folded proteins, participate in regulated degradation of misfolded proteins, protect the cytoskeleton, decrease the damage induced by oxidative stress, and participate in the regulation of certain enzymes as well as apoptosis^6–8^. Ten sHSPs, named HSPB1-HSPB10, are encoded by the human genome^6,9,10^. As their name suggests, members of this family have a relatively low monomeric molecular mass, ranging between 16.8 and 28.3 kDa in humans. These proteins typically form highly polydisperse homo- and heterooligomers that vary in the number (from dimers to 24-mers and more) of component subunits^6,7^. All sHSPs demonstrate a highly conserved tertiary structure consisting of the hallmark α-crystallin domain (ACD), an 80–100 residue β-sandwich structure, and the variable and poorly structured N- and C-terminal domains^6–8^. The ACD has a propensity to dimerize both in isolation and in the context of a full-length protein, while the highly mobile N- and C-terminal domains are mainly responsible for the interdimer interactions driving the formation of the sHSP oligomers^11,12^.

Three main subtypes of the CMT disease have been described: a demyelinating form termed CMT1, axonal form CMT2, and an intermediate form^1,2,13^. The three forms can be discriminated by the measurement of the motor nerve conduction velocity. Mutations in HSPB1 were found in patients with the CMT2 disease, which corresponds to 10–12% of all CMT cases^14^. Specifically, depending on a particular cohort studied, 7–20% of patients with CMT2 or the clinically related distal hereditary motor neuropathy were reported to have mutations in HSPB1^13,15,16^. A total of about twenty mutations scattered along the sequence of HSPB1 have been described^5,17^. Due to the complex oligomerization properties of this sHSP as well as the involvement of multiple cellular partners, the effect of mutations on HSPB1 activity, and by extension their deleterious effect in patients, has proven challenging to explain mechanistically^17^. However, the *in vitro* physico-chemical properties of a number of recombinant HSPB1 mutants in the N- and the C-terminal domains^18^, as well as a few mutants within the ACD have been investigated^19,20^.

**Figure 1:**
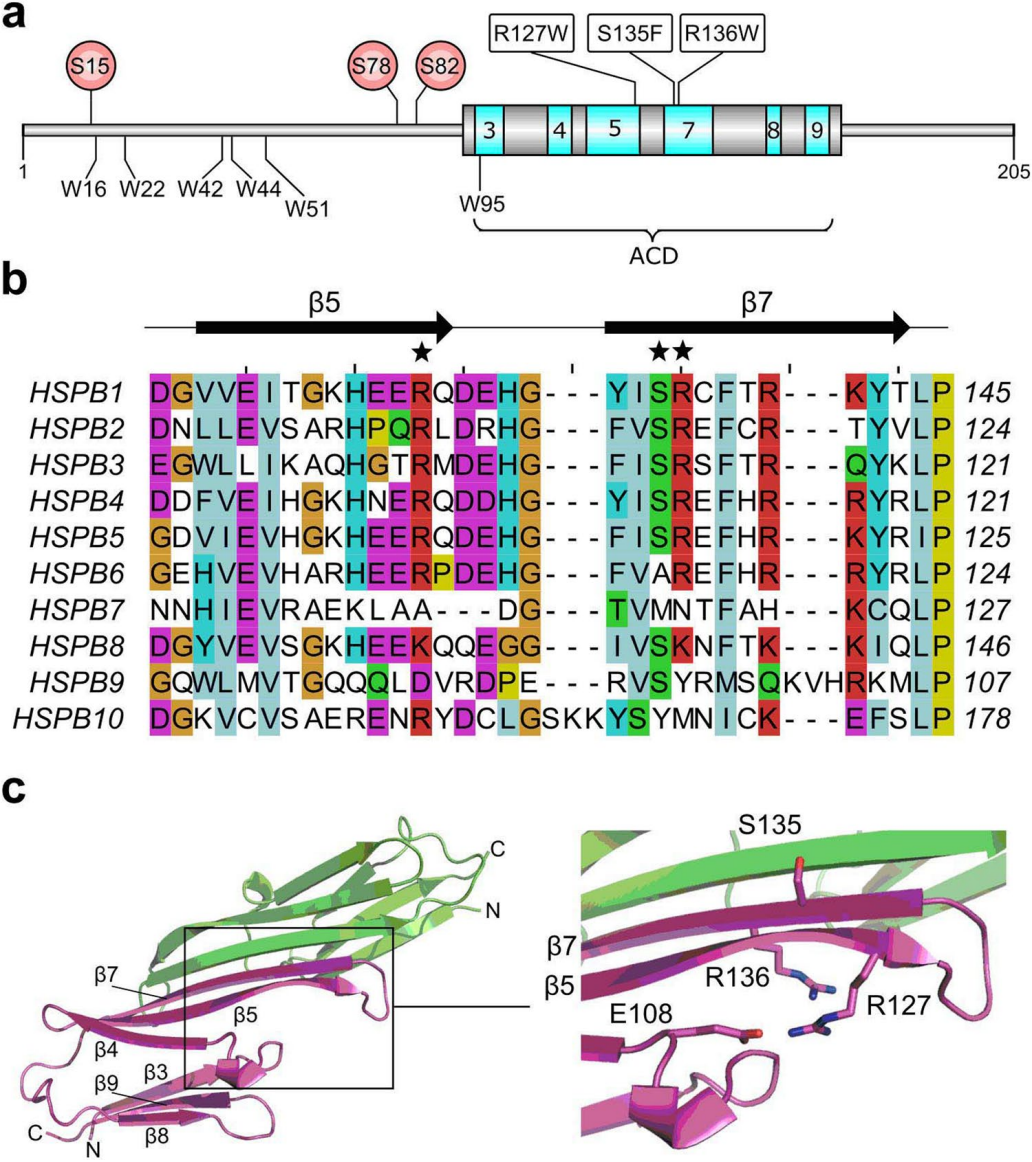
Structural localization of the studied HSPB1 mutations. **(a)** Domain architecture of HSPB1. The core β-strands of the ACD are colored in cyan, their positions and numbering are based on the crystal structure (PDB code 4MJH). Positions of the three MAPKAPK-2 phosphorylation sites are shown as red circles. The six native tryptophan residues are shown below the cartoon. Mutations under study are indicated. **(b)** Predicted secondary structure-based alignment of the region encompassing the ACD β5 and β7 strands of all human sHSPs. The asterisks above the alignment highlight the position of the three characterized mutants. Residue shading is based on the Clustal X color scheme. **(c)** Mapping of the studied mutants onto the HSPB1 ACD structure. The monomers of the dimeric ACD are represented as ribbons (green and magenta). Insert shows a close-up of the region where the mutations are located. The side chains of the three WT residues are shown in stick style. The residue E108 forming a salt bridge with R127 in some crystal structures is also drawn. The bound C-terminal peptide, present in the solved crystal structure, has been omitted for clarity.

A more systematic understanding of the effect of the autosomal dominant HSPB1 mutations R127W, S135F and R136W^21^, all located in the ACD (Fig. 1), was obtained through studies in both cell culture^22^ and in transgenic mice models^23^. Counterintuitively, these mutants appear to possess higher *in vivo* chaperone activity and are more effective in conferring thermotolerance to cells compared to the wild type (WT)^22^. It was speculated that these mutants are hyperactive due to their increased monomerization resulting in a more effective and tighter interaction with different target proteins^22^. This has led to a model whereby the altered binding properties of these HSPB1 mutants to tubulin and the microtubule deacetylase HDAC6 may affect the stability and remodeling of microtubules, as well as disrupt transport along these cytoskeletal elements. These processes are essential in neurons, which explain the fact that the mutations lead to neurodegeneration^23,24^.

Here we present a first comprehensive analysis focusing on the physico-chemical properties of the HSPB1 R127W, S135F and R136W mutants *in vitro*. Building upon the *in cellulo* characterization mentioned above^22,23^, our analysis seeks to provide a rational link between each particular mutation at the molecular level and its phenotype observed in the cell.

## Results

### Spectral properties and thermal stability of HSPB1 mutants

**Figure 2:**
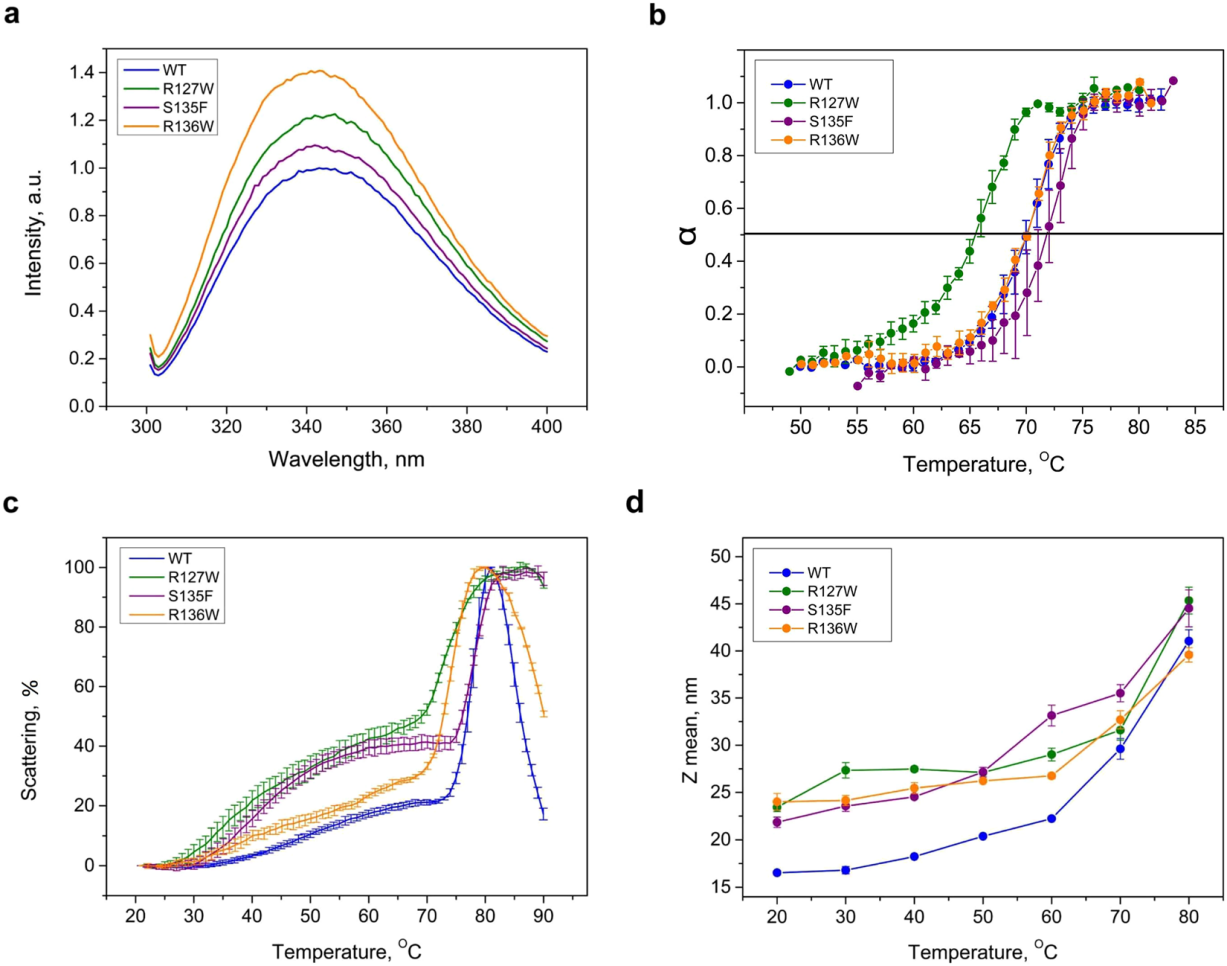
Fluorescence and light scattering properties of the HSPB1 constructs. **(a)** Normalized spectra of fluorescence of the WT HSPB1 and its mutants. **(b)** Completeness of thermal transition, expressed as a fraction of conversion from native to denatured state (α), calculated on the basis of temperature dependence of fluorescence spectra for different HSPB1 species. Data are representative of at least four independent experiments with error bars corresponding to the standard error of the mean. **(c)** Temperature dependence of light scattering. Data are representative of at least three independent experiments with error bars corresponding to the standard error of the mean. The maximal value of light scattering was taken as 100%. **(d)** The effect of temperature on the average particle hydrodynamic diameter determined by DLS. Data are representative of at least four independent experiments with error bars corresponding to the standard error of the mean.

Human HSPB1 contains six Trp residues. Five of these are located in the N-terminal domain which has a high degree of predicted structural disorder, with the remaining residue situated in the β3-strand of the ACD (Fig. 1a). The three HSPB1 mutations under study convert a polar residue to either a Trp or a Phe. In line with this, we have observed higher fluorescence upon excitation at 295 nm for all mutants compared to the WT (Fig. 2a). The fluorescence maxima of all proteins are similar (344 ± 2.5 nm). This value suggests that despite a large number of hydrophobic residues in these constructs the Trp residues are mostly exposed to the solvent.

We have also used Trp fluorescence to analyze the thermal stability of the different HSPB1 mutants (Fig. 2b). Sharp transitions were detected at 69.8 ± 0.8, 65.5 ± 0.5, 71.60 ± 1.1 and 70.1 ± 0.2 °C for the WT and the R127W, S135F and R136W mutants respectively (mean ± SD, N = 4).Thus, only the R127W mutant is less thermostable than the WT.

Self-association of HSPB1 upon heating was previously observed^25^. We have therefore analyzed the temperature-dependent self-association of HSPB1 and its mutants by measuring light scattering at 340 nm (Fig. 2c). A two-phase profile was detected for all four constructs. The first small increase of the light scattering was detected in the range of 30–70 °C and was particularly pronounced for the R127W and S135F mutants. The second much larger increase of light scattering was detected between 70 and 80 °C and, paralleling the transition observed by Trp fluorescence, likely reflects partial protein unfolding followed by aggregation. The data suggest that all three mutants are more prone to temperature-induced association than the WT.

We further probed these temperature-induced structural changes using dynamic light scattering (DLS). All constructs were measured at 0.3 mg/ml, corresponding to a momomer molar concentration of 13 µM and each showed a clear increase of the particle size (hydrodynamic diameter) with increasing temperature (Fig. 2d). At 20–60 °C, the size of the oligomeric species formed by the mutants was distinctly larger than that of the WT. This difference became smaller at higher temperatures where irreversible aggregation was likely dominating the scattering.

### Effect of mutations on the quaternary structure of HSPB1

**Figure 3:**
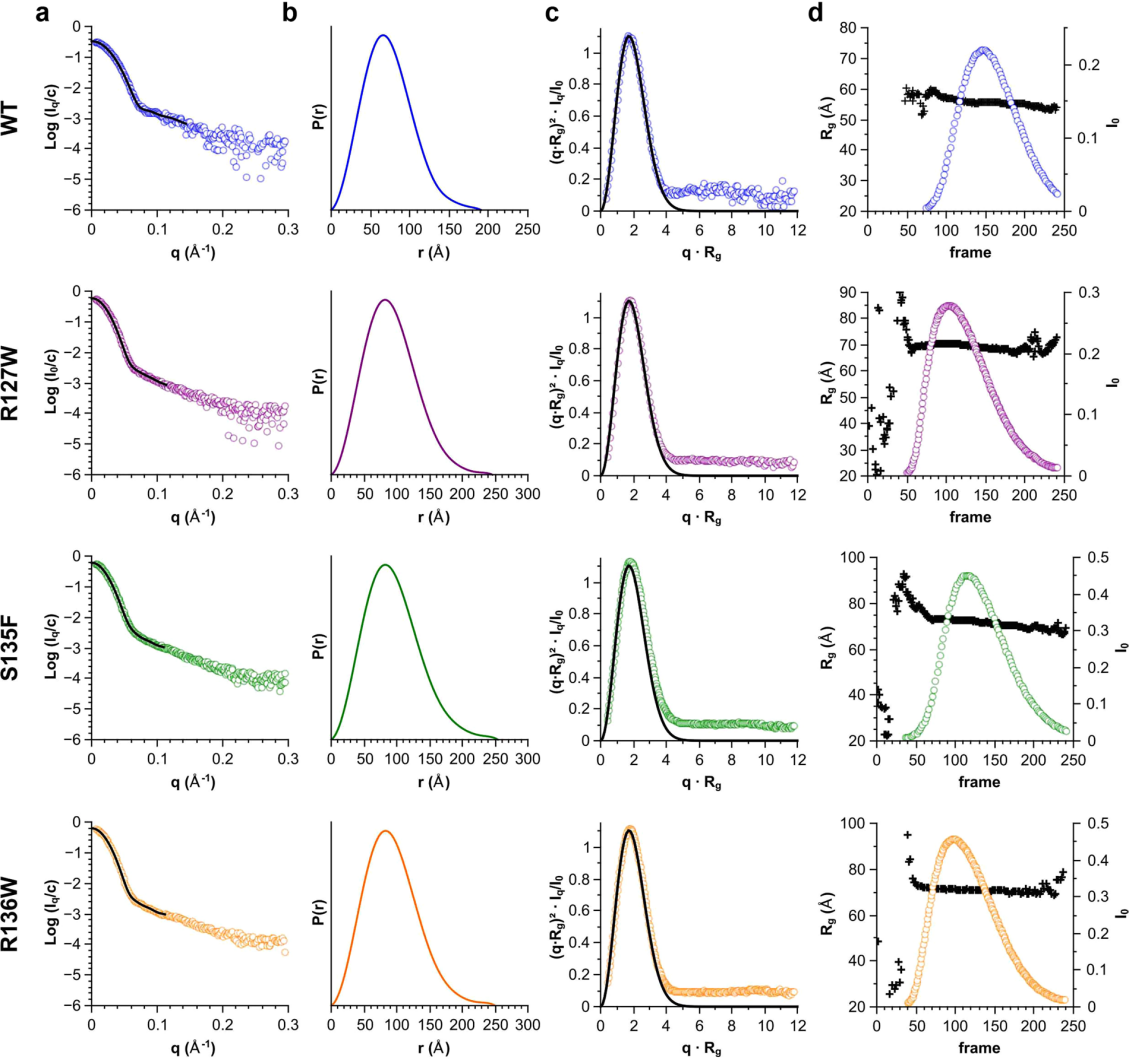
SEC-coupled SAXS analysis of WT HSBP1 and the three ACD mutants. For each construct four plots are shown: **(а)** the averaged scattering curve (circles) and overlayed regularized curve (black line) calculated using data at the elution maxima, **(b)** the corresponding pair-distribution function, **(c)** dimensionless Kratky plot of the same data, and **(d)** the radius of gyration (black crosses, left axis) and normalised forward scattering (circles, right axis) at each elution point. For reference the dimensionless Kratky plot also shows the Guinier approximation of a spherical particle at low angles (black line) plotted using the function f(x) = x² * exp(−x²/3).

While our DLS measurements indicated that the mutants were larger in size than the WT HSPB1 at all temperatures tested, we wondered whether this was a result of an increase in the number of subunits per oligomer, or rather of a more loosely packed arrangement of the component protomers (Fig. 3). To investigate this, we used size-exclusion chromatography (SEC) immediately followed by small angle X-ray scattering (SAXS). At the elution peak WT HSPB1 oligomers had a molecular mass of ~500 kDa corresponding to an average of 23 monomers, whilst all three mutant constructs had a mass of ~900 kDa corresponding to ~40 subunits (Table 1). Concomitant with the increase in mass, the SAXS measurements also revealed larger particle dimensions of the three mutants. For WT HSPB1 the radius of gyration (R_g_) was ~56 Å (Fig. 3d and Table 1). In comparison, the three mutants revealed R_g_ values between 71 and 73 Å. This increase in size is also apparent from the calculated pair distribution functions (Fig. 3b). In particular, the WT had a maximal dimension (D_max_) of ~200 Å whereas all mutants showed a D_max_ of ~250 Å. All pair distributions revealed a fairly symmetric peak, indicative of an approximately spherical particle^26^. In line with this, for all constructs the dimensionless Kratky plots derived from the SAXS data (Fig. 3c) consistently showed a parabolic curve signifying the presence of a globular species. The position and height of the peak maxima were the same for all samples, close to that expected for a compact sphere^27^.

**Table 1:** The size of HSPB1 oligomers as determined by SEC-coupled SAXS.

Construct	Estimated molecular mass*, kDa	Calculated number of subunits	R_g_ (Å)
WT	496	23	55.82 ± 0.04
R127W	880	38	70.36 ± 0.05
S135F	915	40	72.86 ± 0.04
R136W	941	41	71.43 ± 0.74

*Based on comparison to I_o_ of water.

We additionally assessed the particle size for different HSPB1 variants using analytical ultracentrifugation of 0.4 mg/ml solutions. In agreement with the SAXS experiments, the WT HSPB1 had a sedimentation coefficient of 15.8 S, whereas the R127W, S135F, R136W mutants were considerably larger with sedimentation coefficients of 23.1, 20.8 and 24.3 S, respectively. Taken together, these results show that the three HSPB1 mutants studied form nearly spherical oligomers composed of more subunits than the WT protein but with a comparable packing density.

### Concentration-dependent oligomerization

**Figure 4:**
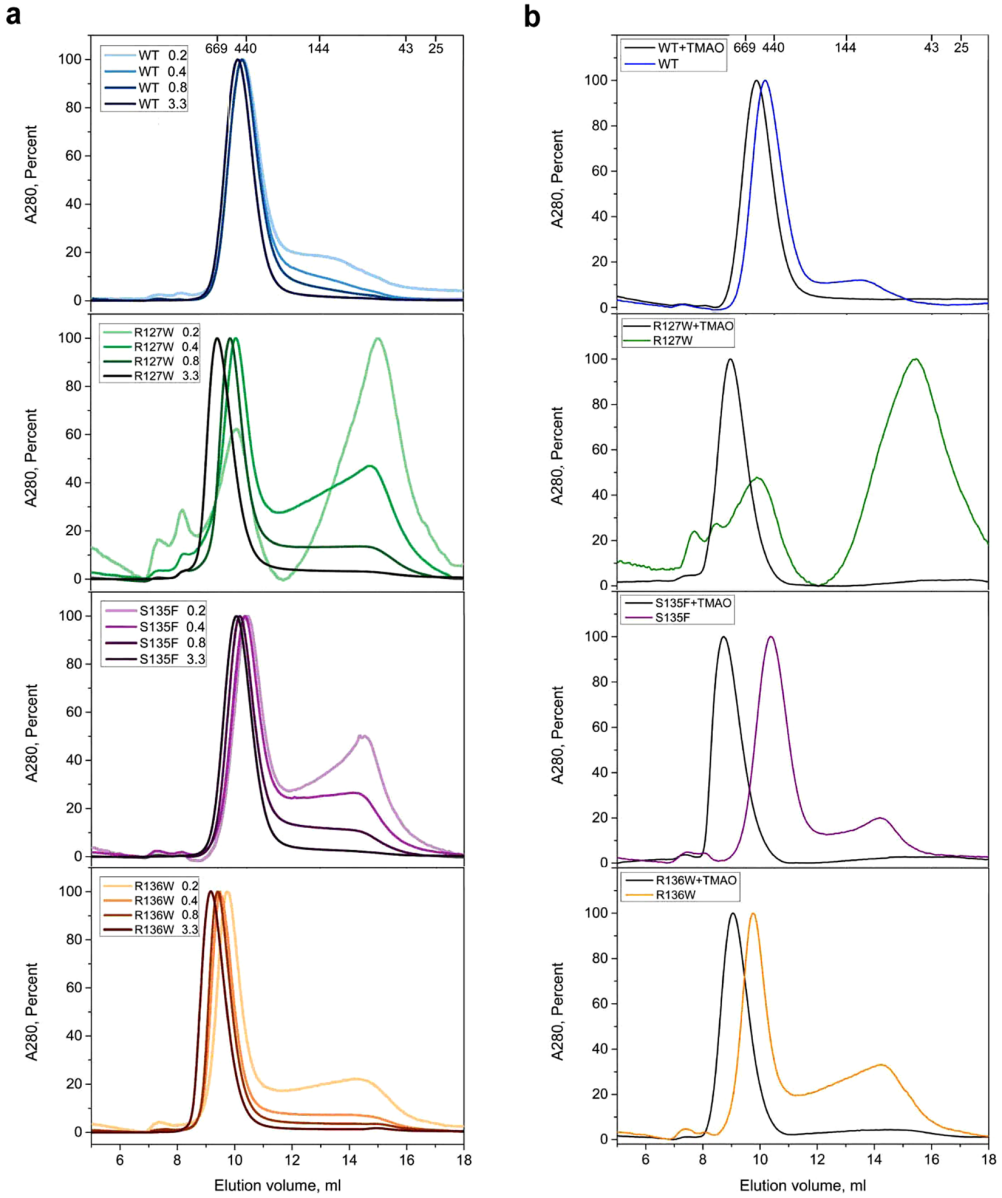
SEC-based analysis of the effects of concentration and crowding on the oligomer size of the HSPB1 variants. **(a)** Effect of concentration. Normalized elution profiles obtained after loading the column with 0.2, 0.4, 0.8 or 3.3 mg/ml (corresponding to 9, 18, 36 and 145 µM monomer molar concentration, respectively) are presented. **(b)** Effect of the crowding agent addition. The proteins were loaded in the absence (colored lines) or presence of 0.5 M TMAO (black lines). Normalized elution profiles obtained after loading the column with 0.2 mg/ml of the protein are presented. Molecular mass calibration is shown above each profile.

A previous report suggested that mutations in the ACD of HSPB1 cause dissociation of the oligomers^22^, but in our SEC-SAXS experiments no smaller entities were observed. As relatively high protein concentrations (5 mg/ml or 220 µM monomer molar concentration) had been loaded on the column to obtain a good SAXS signal, we further addressed this issue using analytical SEC alone (Fig. 4a). The UV-based chromatogram of the WT HSPB1 indicated that under the conditions used the protein formed stable oligomers, with an apparent molecular mass of 550–600 kDa, which dominated the profile even at the lowest loaded protein concentration of 0.2 mg/ml (9 µM). In contrast, the oligomeric state of the mutants was strongly dependent on concentration. For instance, at the lowest concentration the R127W mutant formed two distinct peaks with apparent molecular masses of 500–520 kDa and 60–65 kDa respectively. An increase in the concentration resulted in a decrease in the height of the second peak. At the same time, an increase of the first peak was observed, while the peak maximum shifted from 700 to 830 kDa. A similar behavior was observed for the S135F mutant where an increase of the loaded protein concentration was accompanied by the disappearance of the second peak (apparent molecular mass 75–85 kDa) and increase of amount and size of the larger species (550–620 kDa). The R136W construct had the strongest oligomerization propensity of all three mutants, closely resembling the WT. Only at a loading concentration of 0.2 mg/ml (9 µM) did this mutant form resolvable small oligomers (80–90 kDa), with the large species dominating. At 3.3 mg/ml (145 µM), the large species peak corresponded to an apparent mass of 950 kDa.

Thus, all three mutants have a tendency to dissociate at low concentrations. However, it should be reminded that in a highly crowded cellular environment these proteins may be present in an effective concentration of ~80–350 mg/ml^28^. To evaluate the effect of macromolecular crowding on the oligomeric distribution of the HSPB1 variants we have repeated the SEC runs in the presence of 0.5 M TMAO, using a single loading concentration of 0.2 mg/ml (9 µM) for each construct (Fig. 4b). For the WT, which eluted as a single peak with and without crowding reagent, the TMAO addition induced an increase of ~90 kDa in the apparent molecular mass. For the three mutants, the addition of TMAO resulted in the disappearance of the small molecular mass species and in the appearance of a ~1000 kDa peak.

### Effect of phosphorylation

**Figure 5:**
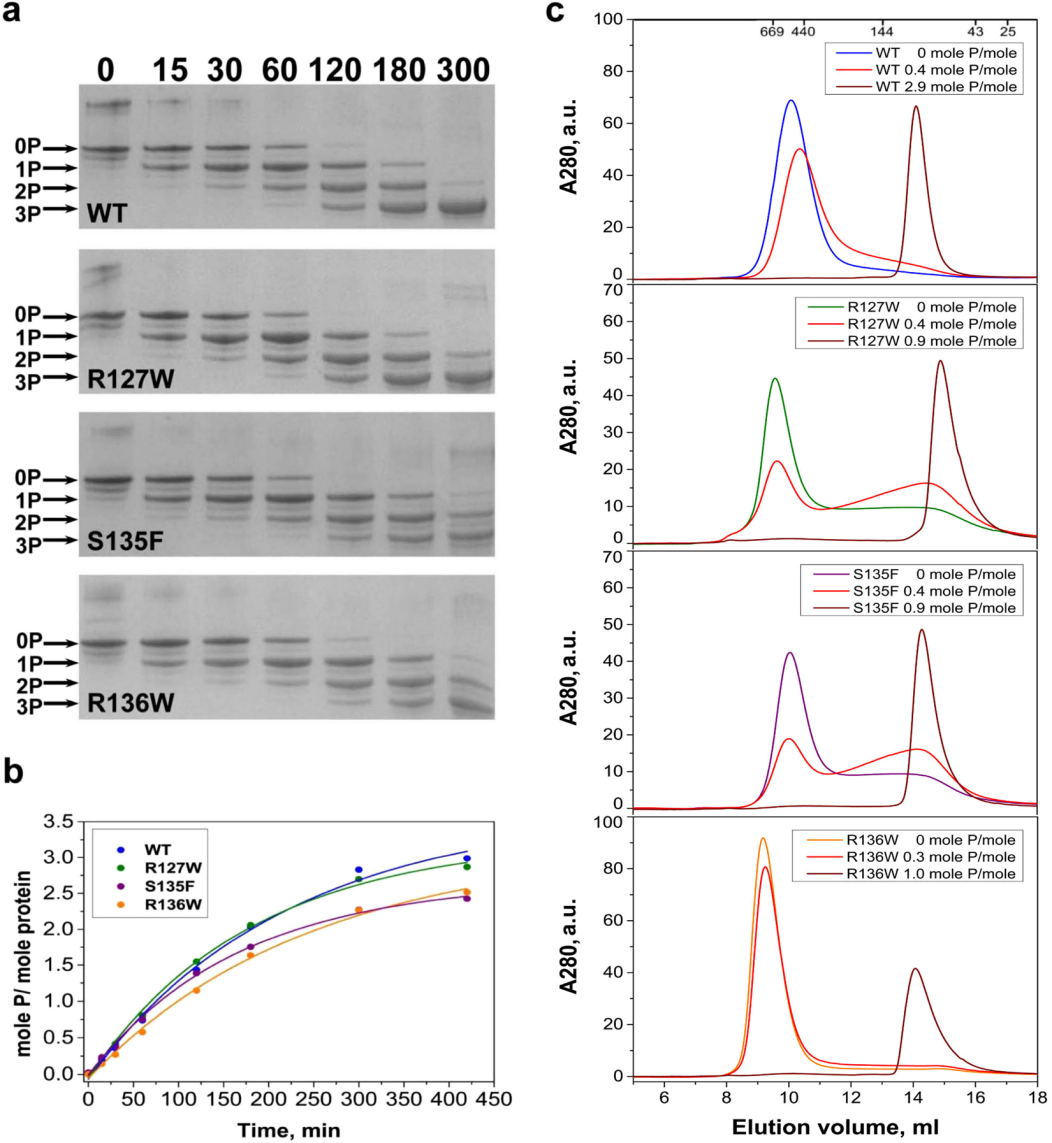
Effect of *in vitro* phosphorylation of HSPB1 and its mutants by MAPKAPK-2. **(a)** Urea gel electrophoresis after different times of incubation with activated MAPKAPK-2. Positions of unphosphorylated, mono-, di- and triphosphorylated species are indicated by arrows. The time of incubation (in min) is indicated above the gel. **(b)** Kinetics of phosphorylation based on the data from the previous panel. **(c)** Analytical SEC of HSPB1 and its mutants before phosphorylation (differently colored lines) and after phosphorylation to the level indicated (shades of red).

Phosphorylation and phosphomimicking mutations are known to affect the quaternary structure of HSPB1 and its chaperone-like activity^29^. Here we have analyzed the effect of the mutations on the susceptibility of HSPB1 for phosphorylation by MAPKAP kinase 2 (MAPKAPK-2), as well the effect of phosphorylation on the oligomeric state. There are three potential sites in the N-terminal domain of human HSPB1, S15, S78 and S82 (Fig. 1a), that are phosphorylated by MAPKAPK-2 *in vivo*^30^. Prolonged incubation with recombinant MAPKAPK-2 was accompanied by a nearly complete phosphorylation, at all three sites, for the WT HSPB1 and the R127W mutant (Fig. 5a,b). At the same time the S135F and R136W mutants were phosphorylated at a marginally lower rate, and the extent of their phosphorylation was slightly lower than for the WT protein.

To assess the effect of phosphorylation on the oligomeric state of different HSPB1 constructs analytical SEC was again employed using a loading concentration of 0.7–1.0 mg/ml (31–44 µM monomer molar concentration) (Fig. 5c). A low level of phosphorylation (0.3–0.4 mole phosphate per mole of protein) did not dramatically affect the oligomeric state of the WT or the R136W mutant. Similar levels of phosphorylation of the R127W and S135F mutants were accompanied by a significant decrease in the quantity of the large oligomeric species and a simultaneous increase of the small oligomer fraction. Phosphorylation of all constructs at 0.9–1.0 mole phosphate per mole of protein or above resulted in a complete dissociation of the large oligomers into smaller species. These species had an apparent molecular mass of 80–85 kDa for the S135F and R136W mutants, similar to 85–90 kDa species observed for WT HSPB1 (Fig. 5c). Phosphorylation of R127W to the same level yielded smaller oligomers with an apparent molecular mass of 60–65 kDa. We conclude that phosphorylation at a single site in each HSPB1 variant already induces the dissociation of the large oligomers.

### Heterooligomeric complexes of HSPB1 and HSPB6

**Figure 6:**
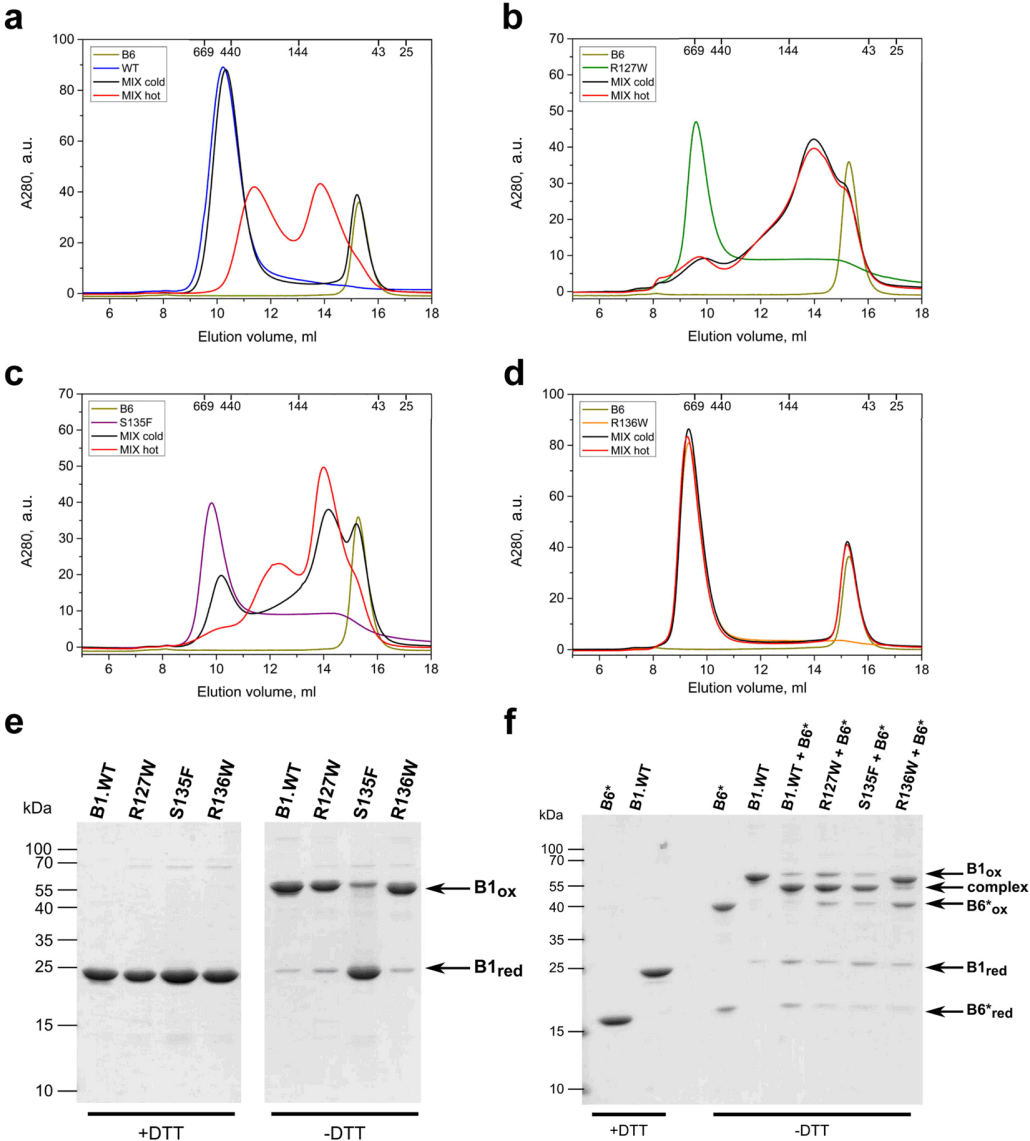
Interaction of the WT HSPB1 **(a)** and its R127W **(b)**, S135F **(c)** and R136W **(d)** mutants with HSPB6 analyzed using SEC. Elution profiles for the four HSPB1 variants alone (differently colored lines), elution profile of isolated WT HSPB6 (green line), elution profiles of the equimolar mixtures of HSPB1 species and HSPB6 preincubated at 4 °C (black line) and the same mixtures preincubated at 45 °C for 1 h (red line) are presented. **(e)** Disulfide cross-linking analysis of HSPB1 variants following an overnight dialysis into a buffer with or without DTT. Non-reducing SDS-PAGE with Coomassie staining was used. **(f)** Similar analysis of heterooligomers formed by the different HSPB1 constructs and the HSPB6 C46S/E116C double mutant (HSPB6*). An equimolar amount of each HSPB1 variant was mixed with HSPB6* and incubated at 37 °C overnight in the presence of DTT. These samples were then further dialyzed at 20 °C for 24 h into a buffer without reducing agent (-DTT). The reduced HSPB6* and WT HSPB1 (+DTT) have also been loaded as controls.

Different members of the mammalian sHSP family are prone to form heterooligomers within the cell^31^. The subunit content and composition as well as the activity of the mixed entities often differs from those of the corresponding sHSP homooligomers^32–34^. Human HSPB1 in particular has been shown to preferentially interact with HSPB6 (HSP20), forming oligomeric species composed of heterodimers^34–36^. To explore the ability of various HSPB1 mutants to form heterooligomeric complexes with HSPB6, we preincubated an equimolar mixture of two sHSPs either at 4 °C or 42 °C and analyzed the composition of the resultant mixture by SEC. As reported earlier^35^, the mixture of WT HSPB1 and HSPB6 formed two main heterooligomeric species with apparent molecular masses of 90–100 and 280–300 kDa respectively (Fig. 6a). These complexes were formed only upon incubating the mixture at a higher temperature, whereas keeping the sample at 4 °C resulted in little to no subunit exchange between these two sHSPs. The R127W mutant readily formed heterooligomeric complexes with HSPB6 even at 4 °C, whereby the two proteins easily exchanged subunits predominantly yielding smaller species peaking at ~90–100 kDa (Fig. 6b). The S135F mutant was also able to exchange subunits with HSPB6 at 4 °C. At high temperature, this mutant preferentially formed heterooligomeric complexes of 90–100 kDa, although large heterooligomers of 280–300 kDa were also detected (Fig. 6c). Unexpectedly, the R136W HSPB1 mutant was unable to form heterooligomeric complexes with HSPB6 either at low or high temperatures (Fig. 6d). Even with extended incubation at 37 °C overnight we were unable to detect any formation of heterooligomeric complexes between these two proteins (data not shown).

Under non-reducing conditions HSPB1 can readily form disulfide-linked dimers (Fig. 6e), resulting from the reaction between the C137 residues in the aligned β7 strands of the two ACDs^32,37^ (Fig. 1). This corresponds to the so-called AP_II_ register of these strands, although two other registers (AP_I_ and AP_III_) have also been observed in crystal structures before^38^. Here we have examined the ability of the mutants to undergo a similar oxidative cross-linking. Following extensive dialysis, the R127W and R136W mutants showed cross-linked dimers, similar in amount to that of the WT HSPB1, as observed by a non-reducing SDS-PAGE (Fig. 6e). In contrast the S135F mutant demonstrated a marked decrease in the formation of disulfide-mediated dimer, suggesting that this mutation affects the dimer interface.

To explore the β7 strand alignment in the heterooligomers formed by mutant HSPB1 with HSPB6, a C46S/E116C double mutant of HSPB6 (termed HSPB6*) was prepared. Since the residue E116 in HSPB6 is in the equivalent position of the β7 strand as the residue C137 in HSPB1, the HSPB6* double mutant forms a disulfide-linked homodimer, or a heterodimer when mixed in an equimolar ratio with the WT HSPB1 under oxidizing conditions^32^. Just like the WT HSPB1, the R127W mutant mainly formed both cross-linked homodimers and heterodimers with HSPB6* (Fig. 6f), suggesting that this mutant does not interfere with preferential association of these two sHSPs nor does it affect the resulting dimer interface. Surprisingly the S135F mutant, previously seen to barely form cross-linked homodimers (Fig. 6e), showed a very similar heterodimer cross-linking pattern to that of the WT HSPB1 and the R127W mutant (Fig. 6f), indicating that, at least in the heterooligomer, this mutant has the capacity to form the canonical dimer interface. Finally, the R136W mutant was unable to form crosslinked heterodimers with HSPB6* (Fig. 6f). This result was in good agreement with the SEC analysis of the equimolar mixture of this mutant with WT HSPB6 (Fig. 6d), and signifies that although this mutant can form homooligomers composed of the canonical AP_II_ type dimers, it has lost the ability to heterooligomerize with HSPB6.

### Chaperone-like activity

**Figure 7:**
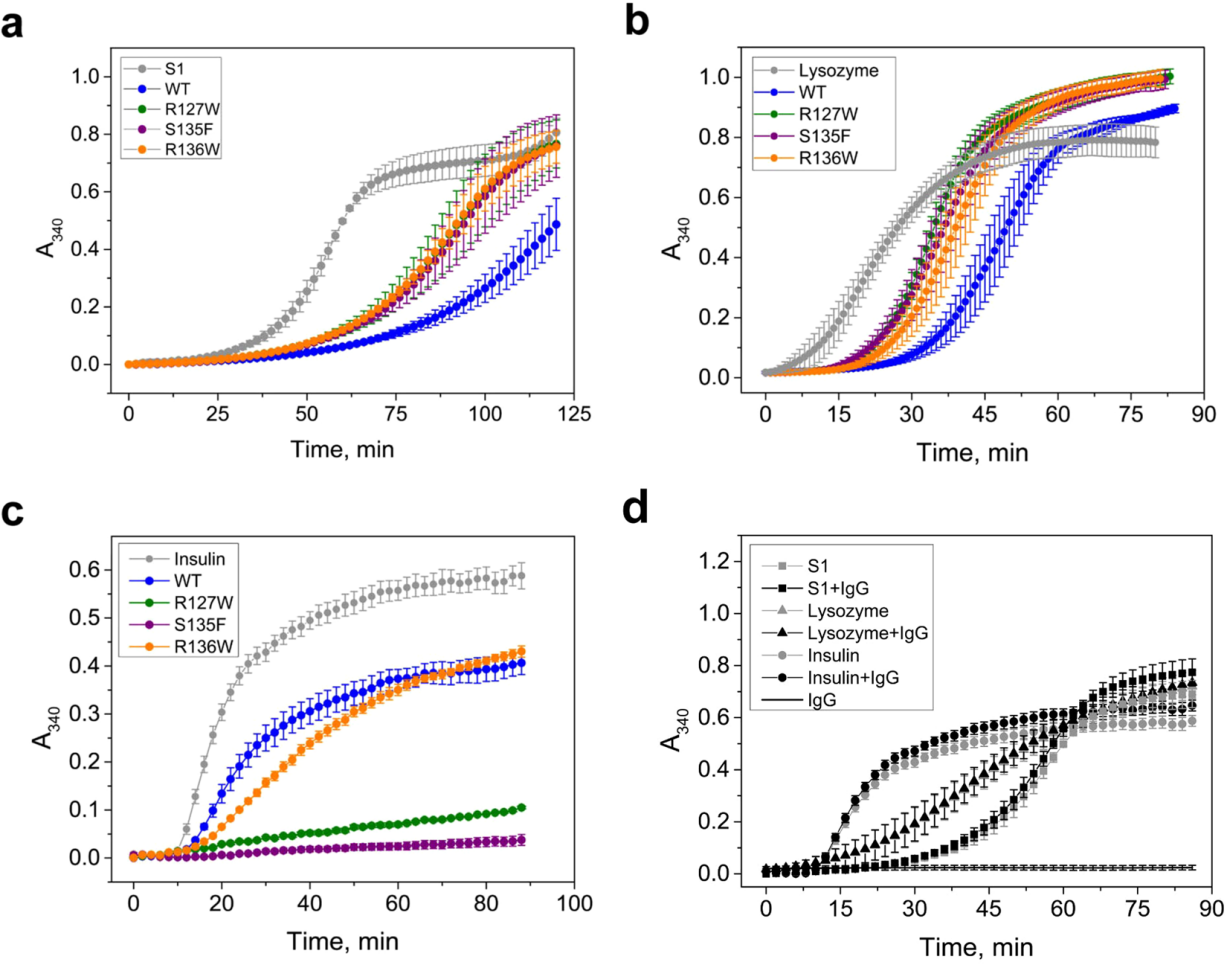
Chaperone-like activity of the WT HSPB1 and the three ACD mutants. Subfragment S1 of skeletal muscle myosin **(a)**, chicken egg-white lysozyme **(b)** and human insulin **(c)** were used as model substrates. In each panel, aggregation curves for the substrate alone and in the presence of the labeled HSPB1 construct are presented. Data are representative of at least four independent experiments with error bars corresponding to the standard error of the mean. **(d)** Negative control experiment showing the aggregation of the three used substrates in the presence and absence of a mouse monoclonal antibody IgG1.

Several model protein substrates were used to analyze the chaperone-like activity of WT HSPB1 and its mutants. The WT protein effectively retarded and decreased the rate of thermal aggregation of the S1 fragment of myosin (Fig. 7a). With this substrate, the chaperone-like activity of the HSPB1 mutants was lower than for the WT protein but similar across all three mutants (Fig. 7a). Comparable results were obtained with a reduction-induced aggregation of lysozyme (Fig. 7b). Here the WT HSPB1 was also more potent than any of the mutants, all of them showing similar activities. Conversely, using insulin as a substrate, the R127W and S135F mutants were more effective than the WT and R136W in preventing DTT-induced aggregation (Fig. 7c). Thus depending on the nature of the model substrate and protein concentration the analyzed mutants were less or more effective than the WT in preventing aggregation. To ensure that the observed protection in the presence of HSPB1 variants was due to their chaperone-like activity rather than simply a crowding effect, control experiments involving a mouse monoclonal antibody IgG1 were performed. In stark contrast with the HSPB1 variants, the presence of IgG1 did not result in any significant change in the aggregation behavior of each of the three substrates. On its own IgG1 did not show any measurable aggregation (Fig. 7d).

## Discussion

Here we have analyzed the effect that three autosomal dominant mutations of human HSPB1 linked to the CMT2 disease have on the physico-chemical properties of this sHSP. The R127W mutation is located in the β5 strand of the ACD, while S135F and R136W mutations are in the β7 strand (Fig. 1a). The mutated residues, which demonstrate considerable conservation between different human sHSP orthologues, anchor an equally well conserved 5-residue hairpin connecting these β-strands (Fig. 1b). In some crystal structures residue R127 is pointing away from the dimer interface and is involved in an intramonomer salt bridge with residue E108. In contrast, the side chain of R136 resides at the dimer interface, pointing into the shared groove formed between the two β3 strands^39^ (Fig. 1c). Whilst also at the dimer interface, the side chain of S135 is found on the opposite side of R136 with respect to the common β-sheet.

Previously, an increase in monomerization of the R127W, S135F and R136W HSPB1 mutants was observed upon stable overexpression in the neuronal-derived SH-SY5Y cell line^22^. Here we show that, in isolation and *in vitro*, such monomerization is not oberved. A combination of SEC, DLS, SAXS and AUC indicate that all three mutants, at concentrations above 0.3 mg/ml *i*.*e*. monomeric molar concentrations of 13 µM, form large oligomers with a molecular mass almost double that of the WT protein at physiological temperatures. It is well recognized though that HSPB1 demonstrates a concentration-dependent dissociation^40^. Here we have investigated whether the ACD mutations affected this property. Both the R127W and S135F mutants, and to a lesser extent R136W, have indeed revealed a substantial fraction of smaller oligomers at loading concentrations where the WT predominantly formed large molecular mass oligomers (Fig. 4). However even at the lowest loaded concentration these smaller species had an apparent molecular mass of 60–80 kDa for all constructs. This value is in line with what was previously observed for the WT protein^40^ and likely corresponds to a dimeric or tetrameric species, but not monomers.

Depending on physiological conditions and the tissue type, the cellular content of HSPB1 has been reported to vary between 150 and 2300 ng per mg of the total protein, which is equivalent to a concentration of 0.1–1.0 mg/ml^41–43^. This is range where our data show that some of the mutants can dissociate. However, in the cell HSPB1 exists in a crowded environment with total protein concentrations in excess of 200 mg/ml and in the presence of numerous other macromolecules, which can increase the self-association of the sHSP. Using the crowding reagent TMAO, we have observed that all three mutants formed exclusively large oligomers even at the lowest concentrations that led to dissociation in normal aqueous conditions. Furthermore, the resultant oligomers were even larger than those observed in the absence of TMAO at loading concentrations greater than used in the crowding experiment (Fig. 4).

Taken together our data show that the R127W, S135F and R136W mutants of HSPB1 result in a formation of homooligomers that are significantly larger than those formed by the WT protein. The question arises why Almeida-Souza *et al*.^22^ could detect the very small molecular mass species (~30 kDa) when analyzing the cellular lysates containing the R127W and S135F mutants. Such species where never detected in our experiments. We propose several explanations for this discrepancy. Firstly, in the cell lysates dissociated species of these mutants could interact with partner proteins, stabilizing the small molecular mass entities. Secondly, in the cellular investigation all HSPB1 mutants were singly or double tagged C-terminally, with either a V5 epitope or a FLAG-TEV cleavage site-Protein A sequence^22^. It has been shown that even small N-terminal His tags can affect the quaternary structure of HSPB1^44^. Therefore the addition of these larger tags could affect the structure and properties of WT HSPB1 and its mutants.

In cell culture, particular in the presence of fetal calf serum, HSPB1 has been shown to be highly phosphorylated and this modification is known to induce dissociation of oligomers^45^. Although Almeida–Souza *et al*.^22^ suggested that phosphorylation does not affect the amount of HSPB1 monomerization, we evaluated the ability of the three mutants to be phosphorylated by MAPKAPK-2 and the effect of this post-translational modification on the resultant oligomers. Although the rate and extent of phosphorylation did vary to a mild extent between the HSPB1 variants, at phosphorylation levels of 0.9–1.0 moles of phosphate per mole of protein or above, complete dissociation of the oligomers was observed independent of the particular mutation (Fig. 5c). In all cases, the apparent molecular masses were similar, and correspond to the dimer previously observed for a triple phosphomimicking mutant of HSPB1^46^.

In addition, a number of human sHSPs can form heterooligomeric species with distinct physico-chemical properties from those of the individual homooligomers^33^. HSPB1 in particular has been shown to heterooligomerize with human HSPB6, a sHSP that exists as a dimer when incubated alone in solution^47^. The resultant heterooligomers show a highly polydisperse size distribution, but are all assembled from a heterodimer building block, which suggests a complete subunit exchange at the monomer level^34–36^. Interestingly the SEC profiles of such heterooligomeric species resemble those seen upon size fractionation of HSPB1 and the mutants in whole cell lysates^22^. We therefore examined the effect of the three HSPB1 ACD mutants on their ability to interact with purified HSPB6. SEC analysis showed that both the R127W and S135F mutants readily formed heterooligomers with this sHSP, and that exchange of subunits even occurred at 4 °C, a temperature where WT HSPB1 does not heterooligomerize during the same incubation period (Fig. 6). For both mutants the resultant heterooligomers had a biased size distribution when compared to those formed by the WT protein, preferentially forming smaller entities with molecular mass centered around 100 kDa, a value close to a tetramer. Interestingly, the R136W mutant could not form heterooligomers with HSPB6 even upon extended incubation at high temperature.

**Figure 8:**
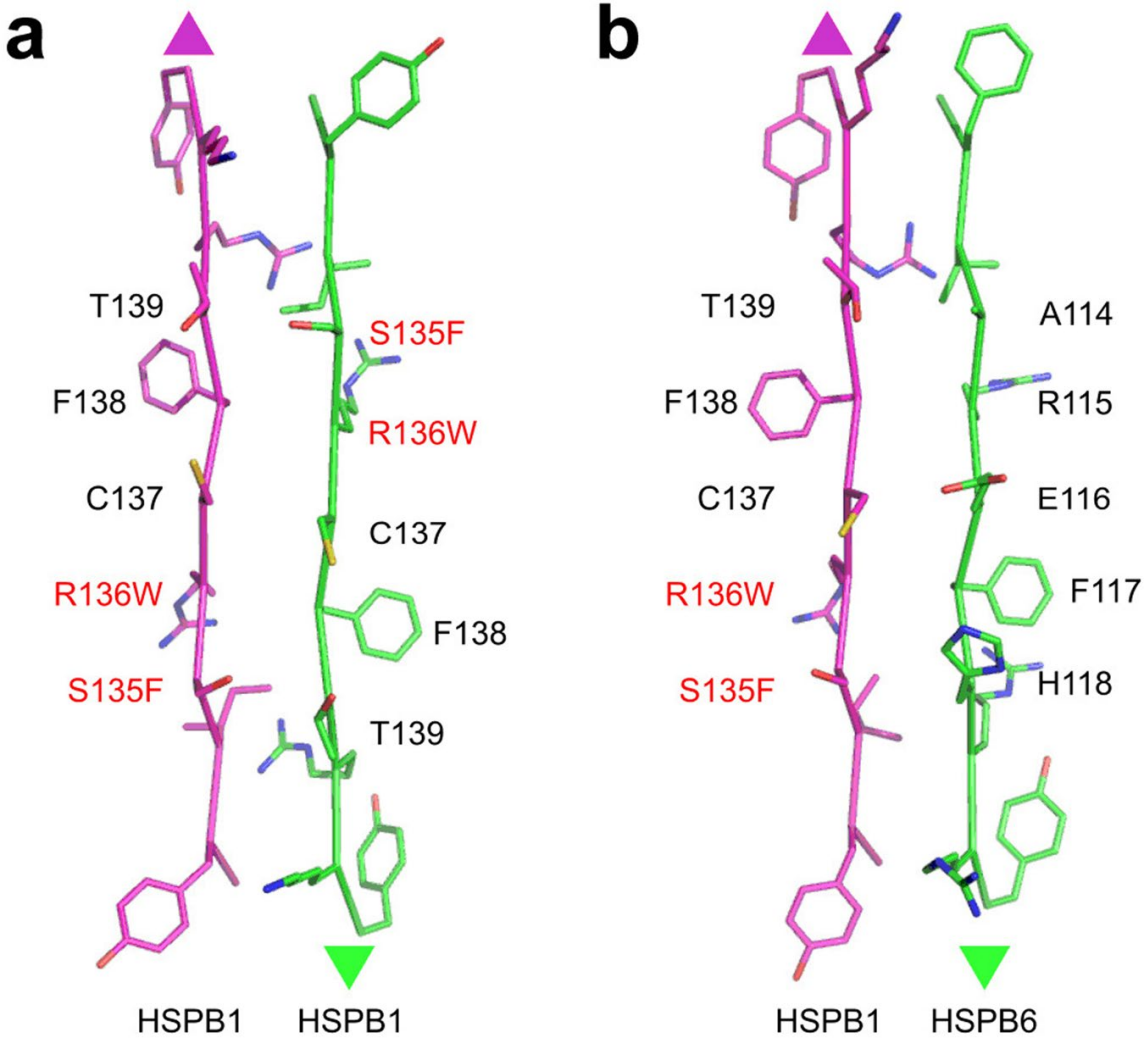
Scheme of the ACD interface residues in the HSPB1 homodimer **(a)** and the HSPB1-HSPB6 heterodimer **(b)**. The residues opposing each other in the β7/β7 dimer interface (AP_II_ register) are shown. The mutated residues characterized in this study are indicated in red.

To further assess the assembly state of the HSPB1 mutants, alone and when mixed with HSPB6, we employed disulfide cross-linking. This exploits the fact that the sole cysteine of HSPB1 located in strand β7 of the ACD can readily oxidize across the dimer interface^32^. For both the R127W and R136W mutants this readily occurred, suggesting that they form a dimer interface similar to that of the WT protein. In contrast, the S135F mutant barely formed cross-linked homodimers (Fig. 6). Upon mixing this particular mutant with a cross-linkable variant of HSPB6, disulfide-linked heterodimers were observed like those formed with WT HSPB1. Assuming an AP_II_ type dimer interface, S135F would sit across from T139 in the homodimer and H118 of HSPB6 in the heterodimer (Fig. 8). Previous modelling suggested that in the first case the two residues would clash and lead to subunit dissociation^22^, but with HSPB6 an equally bulky side chain is present, therefore a more complicated model has to be invoked. In the S135F homodimer the register of the dimer interface could be altered, as seen in a number of X-ray crystal structures^38,48^ or have a different dimer interface altogether. In either case, and with similar effect as suggested by the earlier proposed model, homodimer interaction has to be weaker than that seen in WT HSPB1 resulting in the observable readiness of the S135F mutant to exchange with HSPB6 at low temperatures. In comparison the dimer interface of the R136W mutant, which forms homodimers with the same register as the WT, appears to have the opposite property being more stable. In both the homo and heterooligomer the Trp would sit across a conserved phenylalanine (F138 of HSPB1 and F117 of HSPB6, Fig. 8).

In addition to the structural analyses of the R127W, S135F and R136W mutants we also tested their chaperone-like activity with a number of substrates. Importantly both fluorescence and light scattering studies suggest that only the R127W had reduced thermal stability, but in all cases aggregation occurs only above 65 °C, at temperatures vastly exceeding what is used in the various assays. All mutants possessed lower *in vitro* chaperone-like activity than WT protein when subfragment 1 of myosin or lysozyme were used as model protein substrates (Fig. 7). However, with insulin the mutant proteins had activity that was either comparable or greater than that of WT HSPB1. These conflicting results indicate that the chaperone–like activity is dependent on the nature of the substrate, as well as the conditions of experiment (chaperone concentration, temperature, pH etc). This observation has been repeatedly reported before^35,46,49^. Earlier published data demonstrated that R127W and S135F mutants possess higher *in vivo* chaperone like activity and are more effective in protection of the cell against heat shock^22^. It is difficult to compare the *in vitro* assay results with those obtained *in vivo* for many reasons. For example *in vivo*, as described above, the protein is likely phosphorylated. Such post-translational modification is known to affect the chaperone-like activity depending on conditions and substrate nature^29,46^. At the same time the sHSP is co-existing with many other chaperones, a combination which may prove beneficial. It is also recognized the *in vitro* assays are limiting, and may not reflect the true *in vivo* activity^49^.

In summary, our *in vitro* analysis of the R127W, S135F and R136W mutants of HSPB1 shows that these mutants enhance the size of the HSPB1 homooligomers they form. This is likely the result of changes to intersubunit interactions at the level of the ACD, observed to variable extents for the three mutants. This is specifically highlighted by their capacity to cross-link and form heterooligomeric complexes with HSPB6, and is also reflected in the influence of concentration or phosphorylation on the stability of the homooligomers. Ultimately though, these three mutations lead to the same phenotypic neuropathy. It was recently demonstrated that HSPB1 interacts with many proteins in the cell under heat-shock conditions^49,50^, and that HSPB1 levels are linked to the stability of a number of proteins^6,51^. Although direct protein-protein interactions have yet to be proven, it is feasible that the three mutations alter the residency time of HSPB1 with different targets affecting their turnover and activity. Future studies need to focus on this aspect.

## Materials and Methods

### Proteins

A pET23b construct containing the human HSPB1 cDNA sequence was used by Eurogen (Moscow) for generating expression constructs encoding R127W, S135F and R136W mutants by site directed mutagenesis. The integrity of all expression constructs was confirmed by sequencing. Alternatively, plasmids containing the DNA of three above mentioned mutants were kindly provided by Prof. Vincent Timmerman (VIB department of Molecular Genetics, University of Antwerp, Belgium). The amplified sequences were cloned using the In-Fusion system (Clontech) into pETHSUK, a derivative of published pETHSUL vector containing an additional KpnI cleavage site directly downstream of the SUMO sequence^52^.

Expression of the wild type HSPB1 and its mutants was performed as described earlier^48,53^. The WT and the S135F and R136W mutants were purified by means of ammonium sulfate fractionation followed by anion-exchange and size-exclusion chromatography^47^ or by using subtractive IMAC, anion-exchange and size-exclusion chromatography^48,54^. The properties of proteins purified by the two strategies were indistinguishable. Independent of the construct, the R127W mutant bound irreversibly to the anion-exchange resin, therefore this stage of purification was either omitted or replaced by hydrophobic interaction chromatography using phenyl-sepharose resin^47^. Human HSPB6 and the double mutant C46S/E116C were expressed and purified as described earlier^54,55^. Protein concentrations were determined by absorbance at 280 nm using an extinction coefficient of 40450 M^−1^ cm^−1^ for WT HSPB1 and the S135F mutant, and 45950 M^−1^ cm^−1^ for the R127W and R136W mutants. A value of 9970 M^−1^ cm^−1^ was used for the HSPB6 and its double mutant. All described molar concentrations correspond to the sHSP monomers.

### Fluorescence spectroscopy

Intrinsic Trp fluorescence of the HSPB1 variants was measured in buffer F (20 mM HEPES/NaOH pH 7.5 containing 100 mM NaCl and 2 mM DTT) at 25 °C and at protein concentrations of 0.1 and 0.3 mg/ml. An excitation wavelength of 295 nm (slit width 5 nm) was used and fluorescence was recorded in the range of 300–400 nm (slit width 5 nm). The thermal dependence of fluorescence was measured by gradually increasing the temperature of the sample at a rate of 1 °C/min and measuring fluorescence at 320 and 360 nm (excitation at 295 nm). The temperature of the thermal transition was determined as described earlier^18^ by plotting dependence of fluorescence at 320 nm against fluorescence at 360 nm^56^.

### Temperature dependence of light scattering

The heat-induced increase of light scattering was measured in buffer F at a protein concentration of 0.3 mg/ml. The samples were heated at a rate of 1 °C/min in the range of 20–90 °C. The sample was illuminated at 340 nm and the signal recorded at 340 nm. All measurements of fluorescence and light scattering were performed on Varian Cary Eclipse spectrofluorometer.

### Size-exclusion chromatography

The samples were subjected to size-exclusion chromatography on Superdex 200 HR column equilibrated with buffer S (20 mM Tris/acetate pH 7.6 containing 150 mM NaCl, 0.1 mM EDTA, 0.1 mM PMSF and 15 mM ME). Variable quantities of analyzed proteins (30–500 μg) dissolved in 150 μl of buffer S were loaded on the column and eluted at a flow rate of 0.5 ml/min. The column was calibrated with the following standard: thyroglobulin (669 kDa), ferritin (440 kDa), pyruvate kinase (240 kDa), skeletal muscle glyceraldehydes-3-phosphate dehydrogenase (136 kDa), bovine serum albumin (68 kDa), ovalbumin (43 kDa), chymotrypsinogen (25 kDa) and RNase (13.7 kDa).

### Dynamic light scattering

DLS experiments were performed using a Zetasizer Nano ZS (Malvern). Each protein was diluted to 0.3 mg/ml in 20 mM HEPES/NaOH pH 7.5, containing 100 mM NaCl and 2 mM DTT. Experiments were repeated at 30, 40, 50, 60, 70 and 80 °C. The intensity-weighted value of the mean molecular mass (Z-average) and polydispersity was determined by single cumulant fitting of the decay function.

### Small angle X-ray scattering

For SEC-SAXS experiments, 80 μl of each protein at 5 mg/ml was injected into Shodex KW404-F column equilibrated in 50 mM phosphate pH 7.5, 100 mM NaCl, 2.5 mM DTT with a constant flow-rate 0.2 ml/min and 250 protein frames were collected with an exposure time of 0.75 s and a dead time of 1 s. Buffer averaging, subtraction and processing was performed as described earlier^54^. To generate the average scattering curve the processed curves around the elution maxima were scaled to the curve with the highest I_0_ and averaged using PRIMUS^57^. The intraparticle distance distribution function was solved using GNOM^58^ incorporating the averaged scattering curve data up to q = 0.3 Å^−1^. The dimensionless Kratky plot of the averaged scattering data was generated using the reciprocal space R_g_ and I_0_ values calculated with GNOM. The radius of gyration (R_g_) and forward scattering (I_0_) were calculated at each measured point using buffer subtracted scattering curves that had been averaged by a ten-frame moving average algorithm to improve the signal-to-noise. The forward scattering values are normalized by dividing the values by the concentration of the sample at the peak maximum. As the data was measured on an absolute scale, using water as a reference, the normalized I_0_ maxima for each construct was used to calculate the molecular mass.

### Analytical ultracentrifugation

Protein samples (0.4 mg/ml) in 50 mM phosphate (pH 7.5) containing 150 mM NaCl and 2 mM DTT were subjected to ultracentrifugation (40.000 rpm) in a Spinco E analytical ultracentrifuge as described earlier^19^. The sedimentation coefficients were estimated from differential distribution of sedimentation coefficients [c(s, f/f_o_) versus s] using SEDFIT program^59^.

### Phosphorylation of HSPB1

HSPB1 and its mutants were phosphorylated by MAPKAPK-2 as described earlier^60^. Briefly, all experiments were performed in buffer containing 25 mM HEPES/NaOH, 25 mM β-glycerophosphate, pH 7.5, with 2 mM DTT, 10 mM MgCl_2_ and 400 µM of ATP. p38 kinase was phosphorylated by constitutively activated MKK6 protein kinase. Afterwards activated p38 kinase was used for phosphorylation and activation of MAPKAPK-2. Finally, HSPB1 and its mutants (0.7 mg/ml) were phosphorylated by activated MAPKAPK-2 for different time at 37 °C. In the case when low extent of phosphorylation was desirable we used higher protein concentration (1.0 mg/ml) and reduced the time of incubation with the kinase cocktail. The reactions were stopped by addition of 5 mM EDTA (final concentration) and then analyzed by size-exclusion chromatography and quantitative urea-gel electrophoresis performed on 10% polyacrylamide gel^61^. The extent of phosphorylation was determined either by means of quantitative urea-gel electrophoresis or by liquid scintillation counting. In the last case, phosphorylation of HSPB1 was performed in the presence of γ-^32^P-ATP (2 · 10^6^–5 · 10^6^ cpm/sample).

### Heterooligomeric complexes of HSPB1 and HSPB6

Isolated HSPB1 and HSPB6 were incubated for 30 min at 37 °C in buffer B (10 mM Tris/acetate pH 7.6, containing 0.1 mM EDTA, 0.1 mM PMSF) in the presence of 15 mM DTT to reduce their SH groups. Afterwards the different small heat shock proteins were mixed in buffer S so that the final concentration of each partner was equal to 30 μM per monomer and incubated either at 4 or 42 °C for 1 hr. Alternatively, different small heat shock proteins at final concentration 220 μM per monomer were incubated either at 4 or 37 °C overnight. For the WT HSPB1 and HSPB6 the rate of subunit exchange at 4 °C is negligible, whereas incubation at elevated temperature provides effective subunit exchange^35^. The obtained samples were subjected to SEC under earlier described conditions.

Crosslinked heterooligomeric complexes of HSPB1 and of HSPB6 were formed by mild oxidation leading to formation of disulfide bonds between HSPB1 containing the single Cys137 and the HSPB6 double mutant (C46S/E116C). 50 μM of each protein was mixed in 50 mM sodium phosphate pH 7.5, 100 mM NaCl and 5 mM DTT and incubated overnight at 37 °C. The samples when then dialysed against the same buffer lacking DTT at 20 °C, exchanging the buffer three times over 24 h. Control disulfide cross-linking experiments with the isolated HSPB1 and its mutants were performed by dialyzing each construct against 50 mM sodium phosphate pH 7.5, 100 mM NaCl with and without 5 mM DTT at 20 °C, exchanging the buffer three times over 24 h. The obtained samples were subsequently analyzed on a non-reducing SDS-PAGE containing 15% acrylamide.

### *In vitro* chaperone-like activity

Heat induced aggregation of rabbit skeletal muscle myosin subfragment 1 (S1)^62^ was measured at 42 °C in 20 mM HEPES/NaOH pH 7.0, 115 mM NaCl, 20 mM DTT. Heating of isolated S1 (0.5 mg/ml corresponding to 4.1 µM) was accompanied by time-dependent aggregation. Addition of HSPB1 and its mutants (0.05 mg/ml corresponding to 2.2 µM per monomer) significantly reduced the rate of S1 aggregation. Reduction-induced aggregation of hen egg white lysozyme (Helicon, Russia) was measured as described earlier^60^. All experiments were performed in 50 mM potassium phosphate buffer pH 7.4, 0.1 mM EDTA, 50 mM NaCl at 37°C. Reduction of lysozyme (0.14 mg/ml, 10 µM) by 20 mM DTT was accompanied by its aggregation and addition of HSPB1 or its mutants (0.10–0.15 mg/ml, 4.4–6.6 µM per monomer) reduced the rate of lysozyme aggregation. Similar experiments were performed with insulin (Sigma, USA). Briefly, 0.25 mg/ml (43 µM) of insulin dialysed in 50 mM phosphate buffer pH 7.5, 100 mM NaCl was incubated with each HSPB1 construct at 0.1 mg/ml (4.4 µM per monomer). Aggregation of insulin was induced by addition of 10 mM DTT at 37 °C. Chaperone activity was recorded by measuring the light absorbance at 340 nm. Negative control experiments were done with a mouse monoclonal antibody IgG1 to canine C-reactive protein at a mass concentration equal to that of HSPB1.
